# Matrix Completion and Propagator Method-Based Fast 2D-DOA Estimation with Noise Suppression for Arbitrary EMVS Arrays

**DOI:** 10.3390/s25185769

**Published:** 2025-09-16

**Authors:** Yunzhe Ruan, Weiwei Gong

**Affiliations:** The Electronic Information and Electrical Engineering College, Yangtze University, Jingzhou 434023, China; 2022001380@yangtzeu.edu.cn

**Keywords:** electromagnetic vector sensor, direction-of-arrival estimation, arbitrary array, nonuniform noise, matrix completion

## Abstract

This paper introduces an innovative rapid algorithm for estimating two-dimensional direction of arrival (2D-DOA) with randomly arranged electromagnetic vector sensor (EMVS) arrays under nonuniform noise conditions. The approach begins by forming the covariance matrix of the received signal matrix, after which elements affected by noise are removed based on the characteristics of nonuniform noise to reduce its disruptive effects. Subsequently, the pure covariance matrix is filled using a matrix completion algorithm and then reconstructed into a new matrix. Finally, the signal subspace is extracted by the propagator method (PM) algorithm, and the 2D-DOA is estimated via a method analogous to the Estimation of Signal Parameters via Rotational Invariance Techniques (ESPRIT). Theoretical analyses confirm the high degrees of freedom of the algorithm, low computational complexity, and accuracy of the estimation. Simulation results validate that the proposed algorithm exhibits remarkable resilience against nonuniform noise. When compared with conventional algorithms such as ESPRIT, ESPRIT-like, and improved ESPRIT (IESPRIT), it also shows better performance in terms of estimation speed and accuracy.

## 1. Introduction

In modern communication, radar, sonar, and wireless positioning, array signal processing technology occupies a pivotal position. Among these, direction-of-arrival (DOA) estimation stands as a core task, whose objective is to precisely identify the incoming spatial direction of signal sources [1,2]. This is of great significance for key applications such as target localization, signal separation, and beamforming. With the development of high-resolution algorithms, numerous classic DOA estimation techniques have been introduced and widely applied. Among these approaches, the multiple signal classification (MUSIC) algorithm, a well-established technique in spatial spectrum estimation, has secured a prominent position due to its comprehensive theoretical framework and potential for high-resolution performance [3]. This algorithm estimates signal directions by constructing a spatial spectrum function to perform peak search, achieving high resolution in an ideal white noise environment. However, the MUSIC algorithm has obvious limitations in handling coherent signals and nonuniform noise environments [4], and it is sensitive to array geometry and the number of data samples [5], thereby limiting its practicality.

In contrast, the rotation-invariant technique (ESPRIT) algorithm is generally more efficient. It constructs a rotation-invariant structure of the array, transforming the 2D-DOA estimation problem into a generalized eigenvalue problem, thereby avoiding peak searching [6]. This approach offers advantages in computational efficiency and possesses a certain capability to handle coherent signals—a common challenge in multipath environments that severely degrades the performance of subspace-based methods like MUSIC by causing rank deficiency in the source covariance matrix. It is worth noting that techniques like spatio-frequential smoothing [7] are often employed as preprocessing steps to mitigate this coherence issue, albeit at the cost of a reduced effective array aperture. However, the ESPRIT algorithm itself imposes strict requirements on the array structure for its rotational invariance. A uniform linear array (ULA) serves as its ideal application scenario. Once the array structure deviates from a uniform linear layout, such as in random or irregular arrays, the rotation invariance condition becomes difficult to satisfy, leading to a significant deterioration in algorithm performance. Additionally, although variant algorithms such as ESPRIT-like and improved ESPRIT (IESPRIT) have been proposed to enhance robustness by expanding the array model or introducing weighting mechanisms, their effectiveness is limited [8].

Beyond subspace-based techniques, compressed sensing (CS) methods have recently emerged as powerful alternatives for DOA estimation. By exploiting the inherent sparsity of the spatial signal domain, CS-based methods can effectively resolve coherent sources and operate reliably with limited snapshots [9]. Notably, sparse recovery using an iterative Variational Bayes algorithm has been successfully applied to angle-of-arrival (AOA) estimation, achieving enhanced resolution and robustness [10]. Furthermore, Newton-based optimization strategies and maximum likelihood estimation (MLE) frameworks have also been explored for high-resolution DOA estimation, particularly in coherent source scenarios [11]. These approaches provide valuable complementary perspectives to classical subspace algorithms, thereby enriching the methodological landscape of DOA estimation.

It is widely recognized that the full characteristics of electromagnetic waves at a given location can be characterized by the three orthogonal components of the electric and magnetic fields, enabling a comprehensive analysis of the spatial direction and polarization state of incident electromagnetic waves [12]. An electromagnetic vector sensor comprises six orthogonally oriented sensors, enabling precise capture of the polarization information and propagation direction of incident signals [13]. Unlike traditional scalar sensors, which can only capture signal amplitude, the multi-component design of electromagnetic vector sensors enables simultaneous measurement of the magnitude and direction of both electric and magnetic field components [14]. This unique architecture grants electromagnetic vector sensor arrays significantly higher degrees of freedom (DOF) compared to traditional scalar sensor arrays (SSAs), greatly enhancing their ability to identify signal sources [15]. Additionally, the polarization sensing characteristics of electromagnetic vector sensor arrays confer exceptional versatility, demonstrating outstanding robustness when addressing common challenges in practical applications such as multipath propagation and noise interference. Furthermore, the random array structure, with its flexible deployment and strong adaptability, holds significant application potential in complex or constrained spatial environments [16].

In conventional array signal processing systems, most DOA estimation approaches are predicated on the premise of white Gaussian noise, such as MUSIC, ESPRIT, and their variants [17,18]. This assumption can achieve good estimation accuracy and numerical stability in ideal environments. However, in complex real-world applications, array systems are often affected by factors such as antenna channel gain mismatches, mutual coupling between array elements, hardware impairments in Radio Frequency chains, and external electromagnetic interference, leading to noise exhibiting non-independent and non-identically distributed characteristics, i.e., nonuniform noise [19]. Under these conditions, the noise covariance matrix deviates from the ideal diagonal (white Gaussian noise) form, thereby breaking the orthogonality between the signal and noise subspaces, which severely interferes with subspace-based DOA estimation algorithms [20]. To tackle this issue, a variety of noise suppression strategies have been developed, including spatial cross-covariance methods, covariance differencing methods [21,22], temporal cross-covariance approaches [23], and matrix completion techniques [24]. Spatial cross-covariance approaches suppress noise via subarray partitioning but reduce virtual aperture [25]; temporal cross-covariance methods assume uncorrelated noise between pulses, limiting applicability; high-order cumulant methods require non-Gaussian assumptions and are computationally intensive; and covariance differencing leverages the Toeplitz structure but may introduce ambiguity. While effective in certain scenarios, these methods often lack general robustness in diverse environments.

Existing research has shown that matrix completion techniques can capitalize on the sparse characteristics of the noise covariance matrix, thereby forming a robust system to suppress nonuniform noise [26]. Such methods impose no requirements on presuppositions regarding specific noise features and avoid inducing aperture loss. Based on this, the present study puts forward a rapid 2D-DOA estimation approach tailored for randomly configured EMVS arrays. This method is designed to effectively counteract the interference posed by nonuniform noise to estimation performance, starting with the construction of the received signal covariance matrix and the elimination of noise-polluted elements. Subsequently, the covariance matrix is restored to its complete structure using a low-rank matrix completion method and reconstructed into a data matrix suitable for subsequent processing. Then, the Propagation Operator method is employed to extract the signal subspace, avoiding the traditional feature decomposition step and significantly reducing computational complexity. Finally, a technique analogous to ESPRIT is integrated to realize rapid estimation of the signal’s two-dimensional incident angle. Simulation outcomes indicate that the proposed approach attains higher estimation precision and enhanced robustness in nonuniform noise scenarios while retaining low computational costs, thus verifying the efficacy and advantages of the presented algorithm.

Notations: Mathematical objects are typeset as follows: Vectors are typeset in bold lowercase (m) and matrices in bold uppercase (M). Fundamental operations include transpose ($A^{T}$), conjugate ($A^{*}$), Hermitian transpose ($A^{H}$), inverse ($A^{-1}$), and pseudo-inverse ($A^{†}$). Matrix products are denoted by Kronecker (⊗), Khatri-Rao (⊙), Hadamard (∘), and outer product (⊛). Special matrices include the identity matrix $I_{M}$ and all-ones matrix $1_{M\times M}$. Transformations comprise vectorization (vec(A)) and row-wise diagonalization ($D_{m}\left(A\right)$), while $E{\{}_{▪}\}$ denotes expectation, |·| represents absolute value, ${∥\cdot ∥}_{*}$ denotes the nuclear norm, ${∥\cdot ∥}_{F}$ denotes the Frobenius norm, and vec(·) represents the matrix vectorization operator.

## 2. Problem Formulation

### 2.1. Preliminaries of EMVS

Electromagnetic waves, as transverse waves, have electric and magnetic field components that are mutually orthogonal and oscillate at the same frequency. This characteristic enables the polarization phenomenon of electromagnetic waves to be characterized by changes in the direction of the field vectors. Polarization-sensitive arrays, which respond differently to electromagnetic waves with different polarization states, provide an additional dimension for signal feature extraction. Electromagnetic vector sensors (EMVSs) are a type of polarization-sensitive array element. They feature a co-located structure that combines three mutually orthogonal electric dipoles and three orthogonal magnetic loops. This distinctive configuration allows them to simultaneously measure all six components of electromagnetic fields, specifically three electric field components and three magnetic field components. For *K* incident completely polarized transverse electromagnetic (TEM) plane waves, the ideal (noise-free) output of the EMVS corresponds to the *k*-th wave (k=1,2,...,K), and its polarization characteristics are captured by the response vector $b_{k}$ [27]:

(1) $$b_{k}≜\left[\begin{array}{c} \cos\left(\varphi_{k}\right)\cos\left(\vartheta_{k}\right)\sin\left(\zeta_{k}\right)e^{j\eta_{k}}-\sin\left(\varphi_{k}\right)\cos\left(\zeta_{k}\right)\\ \sin\left(\varphi_{k}\right)\cos\left(\vartheta_{k}\right)\sin\left(\zeta_{k}\right)e^{j\eta_{k}}+\cos\left(\varphi_{k}\right)\cos\left(\zeta_{k}\right)\\ -\sin\left(\vartheta_{k}\right)\sin\left(\zeta_{k}\right)e^{j\eta_{k}}\\ -\sin\left(\varphi_{k}\right)\sin\left(\zeta_{k}\right)e^{j\eta_{k}}-\cos\left(\varphi_{k}\right)\cos\left(\vartheta_{k}\right)\cos\left(\zeta_{k}\right)\\ \cos\left(\varphi_{k}\right)\sin\left(\zeta_{k}\right)e^{j\eta_{k}}-\sin\left(\varphi_{k}\right)\cos\left(\vartheta_{k}\right)\cos\left(\zeta_{k}\right)\\ \sin\left(\vartheta_{k}\right)\cos\left(\zeta_{k}\right) \end{array}\right],$$

and the polarization response vector $b_{k}$ can be decomposed into two subvectors: $e_{k}\in C^{3}$, comprising the first three elements representing the electric field response, and $m_{k}\in C^{3}$, containing the last three elements corresponding to the magnetic field response. The angular parameters are defined within their respective domains: elevation angle $\vartheta_{k}\in \left[-\pi ,\pi \right]$, azimuth angle $\varphi_{k}\in \left[-\pi ,\pi \right]$, auxiliary polarization angle $\zeta_{k}\in \left[0,\pi /2\right]$, and polarization phase difference $\eta_{k}\in \left[-\pi ,\pi \right]$. This vector admits an equivalent factorization expressed as

(2) $$b_{k}=V_{k}g_{k}.$$

where

(3) $$V_{k}≜\left[\begin{array}{cc} \cos\left(\varphi_{k}\right)\cos\left(\vartheta_{k}\right) & -\sin(\varphi_{k})\\ \sin\left(\varphi_{k}\right)\cos\left(\vartheta_{k}\right) & \cos(\varphi_{k})\\ -\sin(\vartheta_{k}) & 0\\ -\sin(\varphi_{k}) & -\cos\left(\varphi_{k}\right)\cos\left(\vartheta_{k}\right)\\ \cos(\varphi_{k}) & -\sin\left(\varphi_{k}\right)\cos\left(\vartheta_{k}\right)\\ 0 & \sin(\vartheta_{k}) \end{array}\right],$$

and

(4) $$g_{k}≜\left[\begin{array}{c} \sin\left(\zeta_{k}\right)e^{j\eta_{k}}\\ \cos(\zeta_{k}) \end{array}\right].$$

Here, $V_{k}\in C^{6\times 2}$ represents the direction-of-arrival parameter matrix and $g_{k}\in C^{2\times 1}$ corresponds to the polarization parameter vector.

The Poynting vector, a key quantity in electromagnetics, describes both the direction and magnitude of electromagnetic energy transfer. In the far-field scenario [28], for transverse electromagnetic (TEM) waves, this vector

- Specifies the wave propagation direction;
- Quantifies the power flux density through its magnitude.

This characteristic allows for accurate DOA estimation through the normalized vector cross-product of electric and magnetic field components:

(5) $$\frac{e_{k}}{∥e_{k}{∥}_{F}}⊛\frac{m_{k}^{*}}{∥m_{k}{∥}_{F}}=p_{k}$$

where $p_{k}$ directly relates to the wave’s propagation direction.

### 2.2. Signal Model

**Figure 1:**
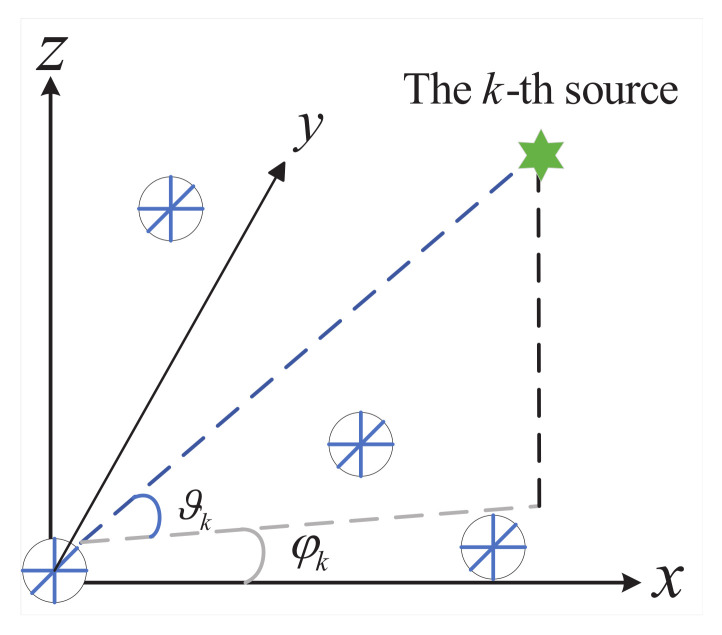
Arbitrarily placed EMVS array.

Considering far-field narrowband plane TEM waves propagating in a homogeneous isotropic medium received by an EMVS array including *M* elements, this work aims to advance the current understanding of the topic. The array features a random geometry, as depicted in Figure 1. The coordinate of the *m*-th EMVS element is denoted by $r={\left[x_{m},y_{m},z_{m}\right]}^{T}$, with the reference element positioned at the coordinate origin $r_{1}={\left[0,0,0\right]}^{T}$. Drawing on the traditional EMVS array model [29], the received signal can be expressed as follows:

(6) $$\begin{array}{cc} y(t) & ≜\left[A⊙B\right]s\left(t\right)+n\left(t\right)\\ \; & =Cs(t)+n(t) \end{array},$$

where $s\left(t\right)\in C^{K\times 1}$ denotes the source vector at time *t*, while $n\left(t\right)\in C^{6M\times 1}$ stands for the nonuniform noise received by the array, and C≜A⊙B, with

(7) $$A≜[a_{1},a_{2},\cdots ,a_{K}],$$

(8) $$a_{k}≜{\left[e^{-j2\pi \tau_{1,k}},e^{-j2\pi \tau_{2,k}},\cdots ,e^{-j2\pi \tau_{M,k}}\right]}^{T},$$

(9) $$B≜[b_{1},b_{2},\cdots ,b_{K}],$$

(10) $$\tau_{m,k}≜\gamma_{m}^{T}g_{k}/\lambda .$$

It is assumed that the noise conforms to a colored Gaussian distribution, featuring a zero mean and a covariance matrix denoted as G [26].

(11) $$E\left\{n\left(t_{1}\right)n^{H}\left(t_{2}\right)\right\}=G\delta (t_{1}-t_{2}),$$

where $\delta {(}_{▪})$ is the unit impulse function; then the covariance matrix is given by

(12) $$\begin{array}{cc} R_{y} & =E\{y\left(t\right)y^{H}\left(t\right)\}\\ \; & =CR_{s}C^{H}+R_{n}\\ \; & =\tilde{R}+R_{n} \end{array}$$

where $R_{s}=E\left\{s\left(t\right)s^{H}\left(t\right)\right\}$ and $R_{n}=E\left\{n{\left(t\right)}^{H}n\left(t\right)\right\}$ are the covariance matrices of the source signal and additive noise. Assuming that the sources are mutually uncorrelated, $R_{s}=\mathrm{diag}\left(\left[\mu_{1},\mu_{2},\cdots ,\mu_{K}\right]\right)$. Using eigenvalue decomposition (EVD), $R_{y}$ can be factorized as follows:

(13) $$\begin{array}{cc} R_{y} & =\sum_{m=1}^{6M}\varrho_{m}u_{m}u_{m}^{H}\\ \; & =U_{s}\Lambda_{s}U_{s}^{H}+U_{n}\Lambda_{n}U_{n}^{H}\\ \; & =U\Lambda U^{H}, \end{array}$$

where $\varrho_{m}$ and $u_{m}$ denote the *m*-th eigenvalue and the *m*-th eigenvector, respectively, and the eigenvalues satisfy $\varrho_{1}\geq \varrho_{2}\geq \cdots \geq \varrho_{K}>\varrho_{K+1}=\varrho_{K+2}=\cdots =\varrho_{6M}$; the signal and noise subspaces are defined as $U_{s}≜\left[u_{1},u_{2},\cdots ,u_{K}\right]$ and $U_{n}≜\left[u_{K+1},u_{K+2},\cdots ,u_{6M}\right]$, and the corresponding eigenvalue matrices are $\Lambda_{s}≜\mathrm{diag}\left\{\varrho_{1},\varrho_{2},\cdots ,\varrho_{K}\right\}$ and $\Lambda_{n}≜\mathrm{diag}\left\{\varrho_{K+1},\varrho_{K+2},\cdots ,\varrho_{6M}\right\}$. Furthermore, we define $U≜[U_{s},U_{n}]$ and $\Lambda ≜\mathrm{diag}\{\varrho_{1},\varrho_{2},\cdots ,$$\varrho_{6M}\}$. In conventional subspace decomposition, the eigenvector matrices $U_{s}$ and $U_{n}$ correspond to the signal and noise subspaces, respectively. It is well known that $U_{s}$ and C share an identical span:

(14) $$U_{s}=CT,$$

where $T\in C^{K\times K}$ represents a reversible and non-singular transformation matrix. Given *L* observed snapshots, the covariance matrix $R_{z}$ can be empirically estimated through

(15) $${\hat{R}}_{y}=\frac{1}{L}\sum_{t=1}^{L}z(t)z^{H}\left(t\right).$$

According to Equation (13), the EVD of ${\hat{R}}_{y}$ is shown as follows:

(16) $$\begin{array}{cc} {\hat{R}}_{y} & =\sum_{m=1}^{6M}{\hat{\varrho }}_{m}{\hat{u}}_{m}{\hat{u}}_{m}^{H}\\ \; & ={\hat{U}}_{s}{\hat{\Lambda }}_{s}{\hat{U}}_{s}^{H}+{\hat{U}}_{n}{\hat{\Lambda }}_{n}{\hat{U}}_{n}^{H}\\ \; & =\hat{U}\hat{\Lambda }{\hat{U}}^{H}, \end{array}$$

where ${\hat{\varrho }}_{m}$, ${\hat{u}}_{m}$, ${\hat{U}}_{s}$, ${\hat{\Lambda }}_{s}$, ${\hat{U}}_{n}$, ${\hat{\Lambda }}_{n}$, $\hat{U}$, and $\hat{\Lambda }$ denote the estimates with respect to $\varrho_{m}$, $u_{m}$, $U_{s}$, $\Lambda_{s}$, $U_{n}$, $\Lambda_{n}$, U, and Λ, respectively. From Equation (12), one can observe that $R_{n}$ possesses a diagonal configuration:

(17) $$R_{n}=\mathrm{diag}\left\{\sigma_{1}^{2},\sigma_{2}^{2},\cdots ,\sigma_{6M}^{2}\right\},$$

where $\sigma_{6(m-1)+n}^{2}$ represents the variance associated with the *n*-th channel of the *m*-th element. In the scenario of white Gaussian noise, the corresponding eigenvalues satisfy $\varrho_{K+1}=\cdots =\varrho_{6M}=\sigma^{2}$, implying uniform noise power across all channels:

(18) $$R_{n}=\sigma^{2}I_{6M}.$$

It has been established that a scaled identity matrix does not alter the eigenstructure associated with the signal subspace. Therefore, conventional subspace-based methods remain valid under white noise environments. But, when the noise becomes spatially nonuniform, then the corresponding covariance matrix deviates from the identity form, which leads to the degradation or failure of many classical algorithms.

## 3. The Proposed Method

### 3.1. Noise Suppression

If a matrix is low-rank, even when certain entries are missing or corrupted by noise, it can still be accurately reconstructed via suitable optimization techniques. As illustrated in Equation (12), the covariance matrix $R_{y}$ exhibits a low-rank structure. Meanwhile, the noise covariance matrix $R_{n}$ corresponding to colored noise typically possesses a sparse profile, as most of its off-diagonal elements are zero or negligible. The combination of the low-rank nature of $R_{s}$ and the sparsity of $R_{n}$ provides an effective basis for mitigating the influence of nonuniform noise. Define Π as the set of index positions corresponding to non-zero elements in the noise covariance matrix $R_{n}$:

(19) Π={(n,n)|n=1,2,⋯,6M}.

Defining an operator $S_{\Pi }\left\{\cdot \right\}$ extracts the matrix elements specified by the index set Π, yielding $S_{\Pi }\left\{R\right\}=\bar{R}\in C^{6M\times 6M}$. The elements of $\bar{R}$ are defined as follows:

(20) $$\bar{R}\left(p,q\right)=\left\{\begin{array}{cc} R(p,q), & (p,q)\in \Pi \\ 0, & (p,q)\notin \Pi \end{array}\right.,$$

Since $R_{n}$ is diagonal, as established in Equation (11), it holds that $R_{n}=S_{\Pi }\left\{R_{n}\right\}$. Noise suppression is achieved by modifying the matrix in Equation (12) to calculate a matrix that is devoid of noise:

(21) $$\begin{array}{cc} {\overset{⌣}{R}}_{y} & =R_{y}-S_{\Pi }\left\{R_{y}\right\}\\ \; & =\tilde{R}-S_{\Pi }\left\{\tilde{R}\right\}. \end{array}$$

This operation effectively mitigates nonuniform noise effects in $R_{y}$ but introduces partial distortion in the signal covariance matrix $\tilde{R}$. Leveraging the intrinsic low-rank characteristic of $\tilde{R}$, we formulate its recovery as a low-rank matrix completion problem:

(22) $$\begin{array}{cc} \; & \min\;\mathrm{rank}\{R\}\\ \; & s.t.\;S_{\Pi }\left\{R\right\}=\tilde{R}. \end{array}$$

This constitutes a non-convex problem, exhibiting the properties of being NP-hard and computationally intractable. To tackle this issue, we adopt a convex relaxation method, substituting the rank function with the nuclear norm:

(23) $$\begin{array}{cc} \; & \min\;{∥R∥}_{*}\\ \; & s.t.\;S_{\Pi }\left(R\right)={\overset{⌣}{R}}_{y}. \end{array}$$

In practice, only an estimate ${\hat{R}}_{y}$ of $R_{y}$ is available, from which we compute ${\overset{⌣}{R}}_{y}$ using Equation (21). To account for the approximation error $∥R_{z}-{\overset{⌣}{R}}_{z}∥$, we introduce an error tolerance ε and reformulate the optimization problem:

(24) $$\begin{array}{cc} \; & \min\;{∥R∥}_{*}\\ \; & s.t.\;∥S_{\Pi }\left(R\right)-{\overset{⌣}{R}}_{z}∥\leq \varepsilon . \end{array}$$

This methodology effectively suppresses nonuniform noise by nullifying the noise-contaminated elements followed by matrix reconstruction. Nevertheless, the current framework fails to account for two critical aspects:

1. The intrinsic structural properties of covariance matrices;
2. The inter-element correlation patterns.

These limitations may induce algorithmic instability and degrade reconstruction accuracy, particularly in low-signal-to-noise-ratio situations.

### 3.2. Matrix Completion Method

Let ρ represent the index set of non-zero elements in $R_{n}$, defined analogously to Equations (21) and (22). Following the methodology of Equation (21), we construct a noiseless covariance matrix:

(25) $${\overset{⌣}{R}}_{y}=R_{y}-S_{\rho }\left\{R_{y}\right\}.$$

The recovery procedure is formulated as follows:

(26) $$\begin{array}{cc} \; & {\min\;∥R∥}_{*}\\ \; & s.t.\;S_{\rho }\left(R\right)={\overset{⌣}{R}}_{y} \end{array}$$

Given an appropriate threshold τ, then

(27) $$\begin{array}{cc} \; & \min_{R}{\;\tau ∥R∥}_{*}+\frac{1}{2}{∥R∥}_{F}^{2}\\ \; & s.t.\;S_{\rho }\left(R\right)={\overset{⌣}{R}}_{y}. \end{array}$$

The corresponding augmented Lagrangian is

(28) $$L\left(R,Y\right)={\tau ∥R∥}_{*}+\frac{1}{2}{∥R∥}_{F}^{2}+\langle Y,S_{\rho }\left({\overset{⌣}{R}}_{y}-R\right)\rangle$$

where $Y\in R^{m\times n}$ is the Lagrange multiplier matrix. The solution incorporates a singular value shrinkage operator $D_{\tau }$, where

(29) $$D_{\tau }\left(Y\right)=UD_{\tau }\left(\Sigma \right)V^{⊺},$$

and

(30) $$D_{\tau }\left(\Sigma \right)=\mathrm{diag}\left(\max(\sigma_{i}-\tau ,0)\right).$$

When τ≥0, the operator $D_{\tau }$ provides the solution to the convex optimization problem:

(31) $$D_{\tau }\left(Y\right)=\underset{V}{\mathrm{argmin}}\left\{\frac{1}{2}{∥V-Y∥}_{F}^{2}+\tau {∥V∥}_{*}\right\}.$$

Then, the iterative solution to the subproblem is derived as follows:

(32) $$\begin{array}{cc} R^{k} & =\underset{R}{\mathrm{argmin}}\left\{{\tau ∥R∥}_{*}+\frac{1}{2}{∥R∥}_{F}^{2}-\langle Y,S_{\rho }\left(R\right)\rangle \right\}\\ \; & =\underset{R}{\mathrm{argmin}}\left\{{\tau ∥R∥}_{*}+\frac{1}{2}{∥R∥}_{F}^{2}-\langle Y,S_{\rho }\left(R\right)\rangle +\frac{1}{2}{∥Y∥}_{F}^{2}\right\}\;(\mathrm{Augmented}\;\mathrm{Lagrangian})\\ \; & =\underset{R}{\mathrm{argmin}}\left\{{\tau ∥R∥}_{*}+\frac{1}{2}{∥R-Y∥}_{F}^{2}\right\}\;(\mathrm{Frobenius}\;\mathrm{Norm}\;\mathrm{Rearrangement})\\ \; & =D_{\tau }\left(Y\right)\;(\mathrm{Singular}\;\mathrm{Value}\;\mathrm{Thresholding}\;\mathrm{Result}), \end{array}$$

and $Y^{k}$ is updated as

(33) $$Y^{k}=Y^{k-1}+\iota_{k}S_{\Delta }\left({\overset{⌣}{R}}_{z}-R\right),$$

where $\iota_{k}>0$ represents the adaptive step size at iteration *k* and τ>0 is a fixed shrinkage threshold. The iterative algorithm terminates when the stopping criterion $∥S_{\Delta }\left({\overset{⌣}{R}}_{z}-R^{k}\right){∥}_{F}\leq \epsilon_{\mathrm{tol}}$ is satisfied, outputting $R^{k}$ as the recovered noise-free covariance matrix, with $\epsilon_{\mathrm{tol}}>0$ being a predefined tolerance level. For the sake of clarity in the subsequent derivations, we continue to denote $\tilde{R}$ as the noise-free signal covariance matrix.

### 3.3. PM Algorithm

The propagator method (PM) algorithm represents a computationally efficient approach to subspace estimation, offering significant advantages over traditional eigenvalue decomposition-based methods. This section details the theoretical foundation of PM and highlights its computational superiority.

(34) $$A=\left[\begin{array}{c} A_{1}\\ A_{2} \end{array}\right],$$

Based on the array manifold model shown in Equation (7), the matrix $A\in C^{M\times K}$ denotes the array manifold matrix. Assuming linear independence of its first *K* rows, the partitioning in Equation (34) leverages the inherent linear structure of the array manifold, establishing the foundation for efficient subspace estimation.

(35) $$A_{2}=P^{H}A_{1},$$

Under the non-singularity condition of $A_{1}$, the propagator operator $P\in C^{K\times (M-K)}$ is defined by Equation (35). This pivotal relationship reveals the linear mapping between submatrices of the array manifold, enabling the avoidance of computationally expensive eigenvalue decomposition.

(36) $$\begin{array}{cc} A & =\left[\begin{array}{c} I_{K}\\ P \end{array}\right]A_{1}\\ \; & ≜QA_{1}, \end{array}$$

As indicated in Equation (36), the array manifold matrix is reconstructed through the product $QA_{1}$ given the following:

1. The columns of A span the signal subspace;
2. $A_{1}$ possesses full rank.

The equivalence between Q and $U_{s}$ establishes that the propagator P contains sufficient structural information to characterize the signal subspace, offering a decomposition-free implementation approach.

### 3.4. Rough 2D-DOA Estimation

This section presents a rigorous formulation for 2D-DOA estimation using EMVS. The core methodology relies on an ESPRIT-like algorithm that extracts polarization ratio parameters $\kappa_{q,k}$ from the estimated signal subspace. The polarization response vector $b_{k}$ is given as follows:

(37) $$b_{k}=b_{k}\left(1\right)\left[\begin{array}{ccccc} 1, & \kappa_{2,k}, & \kappa_{3,k}, & \cdots , & \kappa_{6,k} \end{array}\right],$$

where $\kappa_{q,k}=b_{k}\left(q\right)/b_{k}\left(1\right)$ denotes the polarization ratio for q=2,3,⋯,6. From the array reception model in (5) , we derive the convolutional relationship of normalized polarization responses:

(38) $$\frac{e_{k}}{∥e_{k}{∥}_{F}}⊛\frac{m_{k}^{*}}{∥m_{k}{∥}_{F}}=\frac{\left[\begin{array}{c} 1\\ \kappa_{2,k}\\ \kappa_{3,k} \end{array}\right]}{{∥\left[\begin{array}{c} 1\\ \kappa_{2,k}\\ \kappa_{3,k} \end{array}\right]∥}_{F}}⊛\frac{{\left[\begin{array}{c} \kappa_{4,k}\\ \kappa_{5,k}\\ \kappa_{6,k} \end{array}\right]}^{*}}{{∥\left[\begin{array}{c} \kappa_{4,k}\\ \kappa_{5,k}\\ \kappa_{6,k} \end{array}\right]∥}_{F}},$$

where

(39) $$\begin{array}{cc} {\tilde{e}}_{k} & ≜{\left[1,\kappa_{2,k},\kappa_{3,k}\right]}^{T}\\ {\tilde{m}}_{k} & ≜{\left[\kappa_{4,k},\kappa_{5,k},\kappa_{6,k}\right]}^{T} \end{array}$$

Substituting into Equation (38) yields

(40) $$\begin{array}{cc} \frac{{\tilde{e}}_{k}}{∥{\tilde{e}}_{k}{∥}_{F}}⊛\frac{{\tilde{m}}_{k}^{*}}{∥{\tilde{m}}_{k}{∥}_{F}} & =\left[\begin{array}{c} \cos\left(\varphi_{k}\right)\sin\left(\vartheta_{k}\right)\\ \sin\left(\varphi_{k}\right)\sin\left(\vartheta_{k}\right)\\ \cos(\vartheta_{k}) \end{array}\right]\\ \; & =p_{k} \end{array}$$

This equality demonstrates that the normalized polarization response convolution is equivalent to the directional cosine waveform $p_{k}$, providing the theoretical foundation for estimating azimuth $\varphi_{k}$ and elevation $\vartheta_{k}$ angles. The ESPRIT-like algorithm fundamentally operates by estimating the parameters $\chi_{q,k}$ through signal subspace decomposition. This approach leverages the rotational invariance property inherent in the array manifold, which establishes the following key relationship:

(41) $$\left[A⊙S_{1}\left\{B\right\}\right]\Phi_{q}=A⊙S_{q}\left\{B\right\}$$

where $S_{k}\left\{\cdot \right\}$ denotes the submatrix selection operator (index *k* specifies the subarray) and $\Phi_{q}=\mathrm{diag}\left\{\chi_{q,1},\chi_{q,2},\cdots ,\chi_{q,K}\right\}$ contains the polarization ratios. To operationalize the solution, we define selection matrices and subspace projections:

(42) $$J_{1,p}≜I_{M}⊗i_{6,p}\in C^{M\times 6M}$$

(43) $$U_{1,q}≜J_{1,q}U_{s}$$

Reformulating Equation (41) using these definitions gives

(44) $$J_{1,1}C\Phi_{q}=J_{1,q}C$$

Substituting $C=U_{s}T^{-1}$ into Equation (44) produces $J_{1,1}U_{s}T^{-1}\Phi_{q}=J_{1,q}U_{s}T^{-1}$, which is equivalently expressed as follows: $U_{1,1}T^{-1}\Phi_{q}=U_{1,q}T^{-1}$. This leads to the similarity transformation:

(45) $$T^{-1}\Phi_{q}T=U_{1,1}^{†}U_{1,q}$$

The left-hand side of Equation (45) conforms to the eigenvalue decomposition (EVD) structure, indicating that $\Phi_{q}$ and T can be obtained via EVD of $U_{1,1}^{†}U_{1,q}$. The estimation procedure comprises three steps:

1. Define estimated subspaces ${\hat{U}}_{1,q}≜J_{1,q}{\hat{U}}_{s}$.
2. Perform EVD on $U_{1,1}^{†}{\hat{U}}_{1,2}$ to obtain ${\hat{\Phi }}_{2}$ and $\hat{T}$.
3. Compute remaining polarization ratios:
  (46) $${\hat{\Phi }}_{q}=\hat{T}{\hat{U}}_{1,1}^{†}{\hat{U}}_{1,q}{\hat{T}}^{-1}$$

Let $\kappa_{q,k}$ be the *k*-th diagonal element of ${\hat{\Phi }}_{q}$. Construct the estimation vectors:

(47) $$\begin{array}{cc} {\hat{e}}_{k} & ≜{\left[1,{\hat{\kappa }}_{2,k},{\hat{\kappa }}_{3,k}\right]}^{T}\\ {\hat{m}}_{k} & ≜{\left[{\hat{\kappa }}_{4,k},{\hat{\kappa }}_{5,k},{\hat{\kappa }}_{6,k}\right]}^{T} \end{array}$$

The directional cosine waveform is then estimated as follows:

(48) $${\hat{p}}_{1,k}=\frac{{\hat{e}}_{k}}{∥{\hat{e}}_{k}{∥}_{F}}⊛\frac{{\hat{m}}_{k}^{*}}{∥{\hat{m}}_{k}{∥}_{F}}$$

Finally, the 2D-DOA parameters are recovered through closed-form expressions:

(49) $${\hat{\varphi }}_{1,k}=\arctan\left({\hat{p}}_{1,k}\left(2\right)/{\hat{p}}_{1,k}\left(1\right)\right)$$

(50) $${\hat{\vartheta }}_{1,k}=\pm \arcsin\left(\sqrt{{\hat{p}}_{1,k}{\left(1\right)}^{2}+{\hat{p}}_{1,k}{\left(2\right)}^{2}}\right)$$

where the sign of ${\hat{\vartheta }}_{1,k}$ is determined by $\arccos({\hat{p}}_{1,k}\left(3\right))$. Crucially, the permutation ambiguity induced by T is resolved during the computation of ${\hat{\Phi }}_{q}$, ensuring automatically paired angle estimates.

### 3.5. Refined 2D-DOA Estimation

The proposed algorithm enhances directional cosine waveform estimates by exploiting spatial information in the array manifold matrix A, with particular attention to phase unwrapping challenges. For arbitrary array geometries, the rotational invariance property provides the theoretical basis for our approach:

(51) $$\left[S_{1}\left\{A\right\}⊙B\right]\Psi_{m}=S_{m}\left\{A\right\}⊙B,$$

where $\Psi_{m}≜\mathrm{diag}\left\{\beta_{m,1},\beta_{m,2},\cdots ,\beta_{m,K}\right\}$ with $\beta_{m,k}≜e^{j2\pi (\tau_{1,k}-\tau_{m,k})}$. To operationalize this relationship, we define critical components:

(52a) $$\begin{array}{cc} J_{2,m} & =i_{M,m}⊗I_{6}, \end{array}$$

(52b) $$\begin{array}{cc} U_{2,m} & ≜J_{2,m}U_{s}. \end{array}$$

This rotational invariance can be reformulated into a more computationally amenable form:

(53) $$J_{2,1}C\Psi_{m}=J_{2,m}C.$$

Substituting $C=U_{s}T^{-1}$ yields the fundamental similarity transformation:

(54) $$T^{-1}\Psi_{m}T=U_{2,1}^{†}U_{2,m},$$

which enables eigenvalue decomposition (EVD)-based estimation. Assuming prior estimation of T and $U_{s}$ via ESPRIT-like methods, the phase difference matrix is obtained as follows:

(55) $${\hat{\Psi }}_{m}=\hat{T}U_{2,1}^{†}{\hat{U}}_{2,m}{\hat{T}}^{-1}.$$

Under half-wavelength spacing ($∥\gamma_{1}^{T}-\gamma_{m}^{T}∥\leq \lambda /2$), phase differences directly relate to directional cosines through the linear model:

(56) $$\begin{array}{cc} \mathrm{phase}\{\beta_{m,k}\} & =2\pi (\tau_{1,k}-\tau_{m,k})\\ \; & =\frac{2\pi }{\lambda }\left(\gamma_{1}^{T}-\gamma_{m}^{T}\right)p_{k}. \end{array}$$

This relationship extends to the matrix formulation:

(57) $$\begin{array}{cc} v_{k} & =\mathrm{phase}\left\{{\left[\beta_{2,k},\beta_{3,k},\cdots ,\beta_{M,k}\right]}^{T}\right\}\\ \; & =Fp, \end{array}$$

where the deterministic geometry matrix captures the array configuration:

(58) $$F=\frac{2\pi }{\lambda }\left[\begin{array}{c} \gamma_{1}^{T}-\gamma_{2}^{T}\\ \gamma_{1}^{T}-\gamma_{3}^{T}\\ \vdots \\ \gamma_{1}^{T}-\gamma_{M}^{T} \end{array}\right].$$

**Figure 2:**
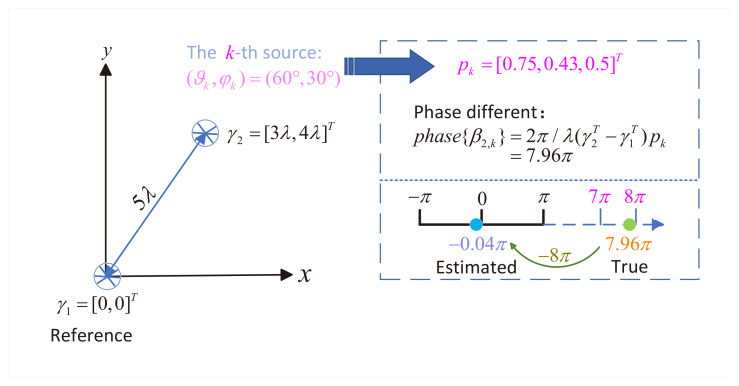
Illustration of phase differences.

This leads directly to the directional cosine estimate:

(59) $${\hat{p}}_{2,k}=F^{†}{\hat{v}}_{k},$$

where ${\hat{v}}_{k}=\mathrm{phase}\left\{{\left[{\hat{\beta }}_{2,k},{\hat{\beta }}_{3,k},\cdots ,{\hat{\beta }}_{M,k}\right]}^{T}\right\}$ and each ${\hat{\beta }}_{m,k}$ represents the *k*-th eigenvalue extracted from the diagonal matrix ${\hat{\Psi }}_{m}$. The relationship in Equation (56) becomes invalid when $∥\gamma_{1}^{T}-\gamma_{m}^{T}∥>\lambda /2$ due to the 2π-periodicity of $e^{jx}$. This periodicity induces phase ambiguity, as demonstrated by a two-EMVS array in the Cartesian coordinate plane, as illustrated in Figure 2. For a source at $\left(60^{\circ },30^{\circ }\right)$ with true $g_{k}={\left[0.75,0.43,0.5\right]}^{T}$, the phase difference $\mathrm{phase}\{\beta_{2,k}\}=7.96\pi$ becomes wrapped to −0.04π after modulo-2π reduction. However, the exponential mapping function would map it to 0.58π by subtracting 2×2π; namely, the estimated phase difference is 0.58π. To accurately fit the directional cosine waveform, we need to determine the true phase difference first. According to the exponential mapping property, we modify Equation (56) to explicitly account for phase periodicity:

(60) $$\mathrm{phase}\left\{\beta_{m,k}\right\}=\frac{2\pi }{\lambda }\left(\gamma_{1}^{T}-\gamma_{m}^{T}\right)p_{k}+z_{m}\cdot 2\pi ,$$

where $z_{m}$ is an integer ambiguity term. Consequently, formulation (57) adjusts to

(61) $${\bar{v}}_{k}=Fp_{k}+2\pi \epsilon_{k},$$

with $\epsilon_{k}={\left[z_{2},z_{3},\cdots ,z_{M}\right]}^{T}$ representing the integer ambiguity vector. Accurate directional cosine estimation now requires resolution of $\epsilon_{k}$. Leveraging the initial estimate ${\hat{p}}_{1,k}$ from the normalized VCP technique in Equation (46), we construct

(62) $${\tilde{v}}_{k}≜F{\hat{p}}_{1,k}.$$

The integer ambiguity vector is then estimated via rounding:

(63) $${\hat{\epsilon }}_{k}=\mathrm{round}\left\{\frac{{\tilde{v}}_{k}-{\hat{v}}_{k}}{2\pi }\right\}.$$

Using this, the true phase vector is recovered as follows:

(64) $${\overset{⌣}{v}}_{k}={\hat{v}}_{k}+2\pi {\hat{\epsilon }}_{k}.$$

The refined directional cosine vector is subsequently computed:

(65) $${\hat{p}}_{3,k}=F^{†}{\overset{⌣}{v}}_{k}.$$

The 2D-DOA are finally obtained through

(66) $${\hat{\varphi }}_{2,k}=\arctan\left({\hat{p}}_{3,k}\left(2\right)/{\hat{p}}_{3,k}\left(1\right)\right),$$

(67) $${\hat{\vartheta }}_{2,k}=\pm \arcsin\left(\sqrt{{\hat{p}}_{3,k}{\left(1\right)}^{2}+{\hat{p}}_{3,k}{\left(2\right)}^{2}}\right).$$

This comprehensive approach resolves phase ambiguities while maintaining the computational advantages of ESPRIT-based methods.

## 4. Algorithm Analysis

### 4.1. Relevant Comments

While the proposed algorithm accommodates arbitrary array geometries, two specific configurations warrant particular attention due to their distinct spatial characteristics. For linear arrays aligned along a single axis, the spatial steering vector depends exclusively on the elevation angle $\vartheta_{k}$. Consequently, the spatial component in Equation (8) modifies to

(68) $$a_{k}≜{\left[e^{-j2\pi x_{1}\sin\left(\vartheta_{k}\right)/\lambda },\cdots ,e^{-j2\pi x_{M}\sin\left(\vartheta_{k}\right)/\lambda }\right]}^{T}.$$

This formulation implies that only elevation angle refinement is feasible through the proposed ESPRIT framework. In such scenarios, fitting matrix *F* consequently adapts to

(69) $$F=2\pi /\lambda \left[\begin{array}{cc} 1 & x_{1}-x_{2}\\ 1 & x_{1}-x_{3}\\ \vdots & \vdots \\ 1 & x_{1}-x_{M} \end{array}\right].$$

Moreover, the phase difference expression similarly simplifies to

(70) $$\mathrm{phase}\;\left\{\beta_{m,k}\right\}=2\pi /\lambda \left(x_{1}-x_{m}\right)\sin\left(\vartheta_{k}\right).$$

Consequently, one can construct an estimated phase difference vector ${\tilde{v}}_{k}$ whose *m*-th element is given by

(71) $${\tilde{v}}_{m,k}≜2\pi /\lambda \left(x_{1}-x_{m}\right)\sin\left({\hat{\vartheta }}_{1,k}\right),$$

where ${\hat{\vartheta }}_{1,k}$ can be roughly determined as per Equation (50). Following the calculations in Equations (63)–(65), one can determine the unambiguous phase difference vector ${\hat{v}}_{k}$ and compute ${\hat{p}}_{3,k}=F^{†}{\hat{v}}_{k}$. Finally, the refined elevation angle can thus be estimated via

(72) $${\hat{\vartheta }}_{2,k}=\arcsin\left({\hat{p}}_{3,k}\left(2\right)\right).$$

For structured geometries such as ULA or URA, the ESPRIT algorithm in [30] presents superior alternatives. Their inherent geometric regularity enables more comprehensive exploitation of rotational invariance properties, potentially yielding enhanced computational efficiency without compromising estimation accuracy.

### 4.2. Flexibility Analysis

As we know, most ESPRIT-based algorithms, including the ESPRIT-like algorithm [31] and the proposed method, are applicable to an arbitrary array geometry. However, the ESPRIT algorithm and the enhanced ESPRIT method (referred to as ‘IESPRIT’) in [30] require a uniform array manifold. Table 1 lists the applicable geometries of the various algorithms. Thus, the ESPRIT-like algorithm and the proposed approach provide a more flexible framework than the traditional ESPRIT and IESPRIT methods.

### 4.3. Identifiability

In ESPRIT-based methodologies, the maximum identifiable quantity fundamentally depends on the maximum rank of the rotational invariance matrix: specifically, the identifiability of ESPRIT-like algorithms is given by $\max\left\{\mathrm{Rank}(\Phi_{q})\right\}=M$; the proposed method’s identifiability is determined by $\max\left\{\mathrm{Rank}\left(\Phi_{q}\right),\mathrm{Rank}\left(\Psi_{q}\right)\right\}$, where $\max\left\{\mathrm{Rank}(\Psi_{q})\right\}=6$; IESPRIT’s identifiability is $\min\left\{\left(M_{1}-1\right)M_{2}-1,\left(M_{2}-1\right)M_{1}-1\right\}$ with $M_{1}+M_{2}=M$; and conventional ESPRIT’s identifiability is $\min\left\{M-1,6\right\}$. Comparative results are summarized in Table 1 , and analysis reveals that for M<6, the proposed method and ESPRIT-like achieve identical identifiability, both exceeding ESPRIT’s capacity, while when M>6, ESPRIT-like demonstrates superior identifiability relative to all benchmarked methods.

### 4.4. Computational Complexity Analysis

A summary of the computational complexity of various algorithms is as follows. Estimating the covariance matrix requires $6^{2}M^{2}L$ complex multiplications, while the eigenvalue decomposition (EVD) involves $O(6^{3}M^{3})$ operations. For the traditional ESPRIT-like method, computing ${\left(J_{1,1}U_{s}\right)}^{†}J_{1,2}U_{s}$ requires approximately $2MK^{2}+O\left(K^{3}\right)$ complex multiplications, and obtaining the rotational matrices ${\hat{\Phi }}_{3},{\hat{\Phi }}_{4},{\hat{\Phi }}_{5},{\hat{\Phi }}_{6}$ contributes another $8MK^{2}+O\left(K^{3}\right)$, yielding a total complexity of approximately $6^{2}M^{2}L+10MK^{2}+O\left(6^{3}M^{3}\right)+O\left(K^{3}\right)$. The ESPRIT method additionally computes ${\hat{\Psi }}_{m}$ for m=2,3,…,M, incurring an extra $6\left(M-1\right)K^{2}+O\left(K^{3}\right)$; thus its overall complexity becomes $6^{2}M^{2}L+10MK^{2}+6\left(M-1\right)K^{2}+O\left(6^{3}M^{3}\right)+O\left(K^{3}\right)$. For the IESPRIT method, constructing and solving the matrix pencil leads to a cost of $O(6^{2}M_{1}^{2}M_{2}^{2}K)$, so the total complexity is $6^{2}M^{2}L+O\left(6^{2}M_{1}^{2}M_{2}^{2}K\right)+O\left(6^{3}M^{3}\right)+O\left(K^{3}\right)$. Compared to these methods, the proposed algorithm also includes the computation of ${\hat{\Psi }}_{m}$, but this additional cost is asymptotically negligible since it is dominated by the $O(6^{3}M^{3})$ term. Notably, the proposed method employs the propagator method (PM) instead of SVD for subspace estimation, significantly reducing computational burden, with the PM step requiring only $O(6MKL+6MK^{2})$ complex multiplications.

### 4.5. CRB

Consider the system model described in Equation (12), where $R_{n}$ is parameterized as Σ(ξ), with $\xi ={\left[\xi_{1},\xi_{2},\ldots ,\xi_{P}\right]}^{T}\in R^{P}$ denoting the vector of unknown real-valued parameters. Reference [32] explicitly presents the theoretical lower bound (CRB) for the joint estimation of 2D-DOA and polarization parameters, which can be expressed as follows:

(73) $$CRB\left(\vartheta ,\varphi \right)=\frac{1}{L}{\left[E-FJ^{-1}F^{T}\right]}^{-1},$$

where

(74) $$\left\{\begin{array}{cc} E & =2ℜ\left\{\left({\tilde{G}}^{H}P_{\tilde{C}}^{⊥}\tilde{G}\right)⊙\left({\left(R_{s}{\tilde{C}}^{H}R_{y}^{-1}\tilde{C}R_{s}\right)}^{T}⊗1_{2\times 2}\right)\right\}\\ F & =2ℜ\left\{\left[\begin{array}{c} V^{T}\left({\tilde{G}}_{d}^{H}P_{\tilde{C}}^{⊥}⊗{\left(R_{y}^{-1}\tilde{C}R_{s}\right)}^{T}\right){\tilde{\Sigma }}^{*}\\ V^{T}\left({\tilde{G}}_{p}^{H}P_{\tilde{C}}^{⊥}⊗{\left(R_{y}^{-1}\tilde{C}R_{s}\right)}^{T}\right){\tilde{\Sigma }}^{*} \end{array}\right]\right\}\\ J & =2ℜ\left\{{\tilde{\Sigma }}^{H}\left(R_{y}^{-T}⊗P_{\tilde{C}}^{⊥}\right)\tilde{\Sigma }\right\}-{\tilde{\Sigma }}^{H}\left(P_{\tilde{C}}^{⊥T}⊗P_{\tilde{C}}^{⊥}\right)\tilde{\Sigma } \end{array}\right.$$

where ℜ{·} denotes the real part operator. The normalized composite steering matrix is defined as $\tilde{C}=R_{n}^{-1/2}C$ with C=A⊙B, where A and B represent the array response matrix and polarization matrix, respectively. The orthogonal projection operator onto the noise subspace is given by $P_{\tilde{C}}^{⊥}=I-P_{\tilde{C}}$, and $P_{\tilde{C}}=\tilde{C}{\tilde{C}}^{†}$. The normalized derivative matrices are constructed as follows: $\tilde{G}=\left[{\tilde{G}}_{d},{\tilde{G}}_{p}\right]$, ${\tilde{G}}_{d}=R_{n}^{-1/2}G_{d}$, and ${\tilde{G}}_{p}=R_{n}^{-1/2}G_{p}$. Let $c_{k}$ denote the *k*-th column of C; the derivative matrices with respect to the estimation parameters are

(75) $$\begin{array}{cc} G_{d} & =\left[\frac{\partial c_{1}}{\partial \vartheta_{1}},\cdots ,\frac{\partial c_{K}}{\partial \vartheta_{K}},\frac{\partial c_{1}}{\partial \varphi_{1}},\cdots ,\frac{\partial c_{K}}{\partial \varphi_{K}}\right]\\ G_{p} & =\left[\frac{\partial c_{1}}{\partial \eta_{1}},\cdots ,\frac{\partial c_{K}}{\partial \eta_{K}},\frac{\partial c_{1}}{\partial \zeta_{1}},\cdots ,\frac{\partial c_{K}}{\partial \zeta_{K}}\right] \end{array}.$$

The received signal matrix is given by ${\tilde{R}}_{y}=\Sigma^{-1/2}R_{y}\Sigma^{-1/2}$. The selection matrix V is defined as follows: $V=\left[\mathrm{vec}\left(e_{1}e_{1}^{T}\right),\mathrm{vec}\left(e_{2}e_{2}^{T}\right),\cdots ,\mathrm{vec}\left(e_{K}e_{K}^{T}\right)\right]$, where $e_{k}$ denotes the *k*-th column of the identity matrix $I_{K}$. The noise covariance derivative matrix is given by $\tilde{\Sigma }=\left[\mathrm{vec}\left({\tilde{\Sigma }}_{1}^{\prime }\right),\mathrm{vec}\left({\tilde{\Sigma }}_{2}^{\prime }\right),\cdots ,\mathrm{vec}\left({\tilde{\Sigma }}_{P}^{\prime }\right)\right]$ with ${\tilde{\Sigma }}_{p}^{\prime }=\Sigma^{-1/2}\Sigma_{p}^{\prime }\Sigma^{-1/2}$ and $\Sigma_{p}^{\prime }=\partial \Sigma /\partial \xi_{p}$. It is worth emphasizing that the CRB represents a theoretical lower bound on the estimation accuracy, serving as a fundamental benchmark for algorithm performance evaluation. In practice, the closer an algorithm’s RMSE is to the CRB, the higher its estimation accuracy can be considered.

## 5. Simulation Results

### 5.1. Simulation Experiments

Root mean square error (RMSE) is a metric for measuring accuracy. The smaller the RMSE value, the closer the predicted value to the actual value, and the higher the accuracy of the model or algorithm. When multiple algorithms are used to solve the same problem, comparing their RMSE values can help determine which model performs better. Algorithms with lower RMSE values typically have higher accuracy. For evaluating the proposed algorithm, the RMSE is adopted:

(76) $$\mathrm{RMSE}=\sqrt{\frac{1}{N_{\mathrm{MC}}K}\sum_{n=1}^{N_{\mathrm{MC}}}\sum_{k=1}^{K}\left[{\left({\hat{\theta }}_{k}^{\left(n\right)}-\theta_{k}\right)}^{2}+{\left({\hat{\phi }}_{k}^{\left(n\right)}-\phi_{k}\right)}^{2}\right]}$$

where $N_{\mathrm{MC}}=500$ Monte Carlo trials, there are *K* targets, and ${\hat{\theta }}_{k}^{\left(n\right)}$ and ${\hat{\phi }}_{k}^{\left(n\right)}$ are estimated angles.

**Figure 3:**
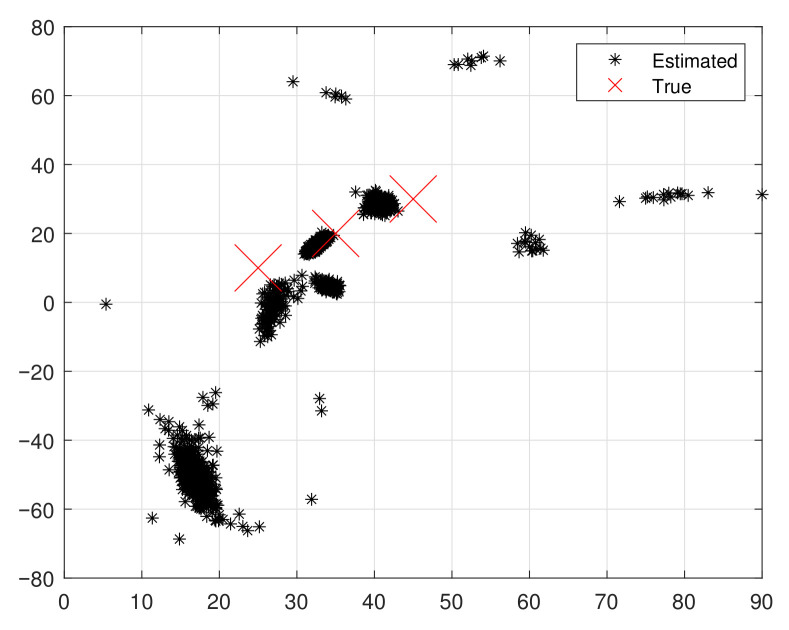
Scatter plot results with noise.

**Figure 4:**
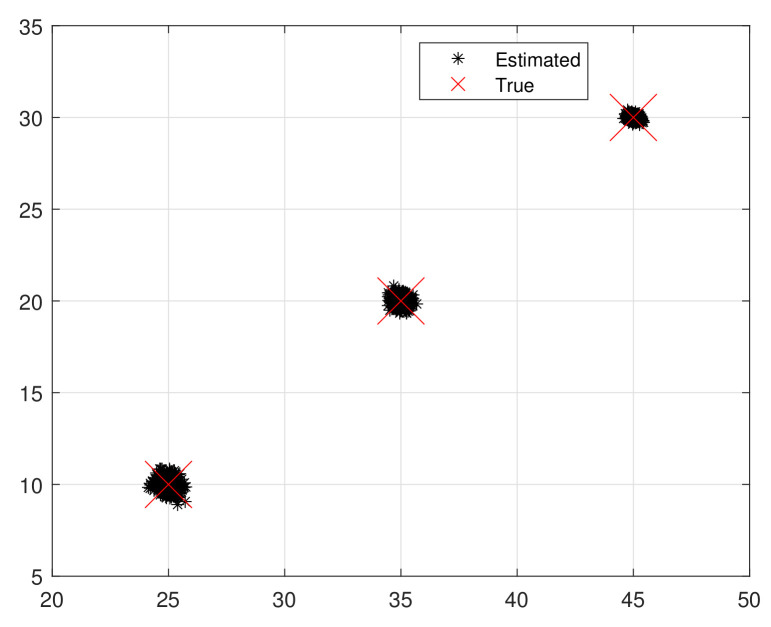
Scatter plot using proposed method. **Note:** To ensure a fair comparison, we suppressed the influence of noise before comparing the RMSE of all algorithms.

The scatter plot in Figure 3 and Figure 4 demonstrates the efficacy of the proposed method, where the *x*-axis represents the range of θ and the *y*-axis denotes the range of ϕ. We consider an EMVS comprising *M* elements and *L* measurements. In the scenario, K=3 TEM signal waves received by the array are corrupted by nonuniform noise. The relevant parameters are set as follows: $\vartheta ={\left[25^{\circ },35^{\circ },45^{\circ }\right]}^{T}$, $\varphi ={\left[10^{\circ },20^{\circ },30^{\circ }\right]}^{T}$, $\zeta ={\left[17^{\circ },42^{\circ },73^{\circ }\right]}^{T}$, and $\eta ={\left[19^{\circ },53^{\circ },-32^{\circ }\right]}^{T}$. The performance metrics are RMSE and average running time (ART). The comparison benchmarks are ESPRIT [33], ESPRIT-like [31], IESPRIT [30], and CRB-L. ESPRIT uses ULA with $M_{1}=2$, $M_{2}=3$ ($M=M_{1}\times M_{2}$, where $M_{1}=2$ denotes the number of array elements along the *x*-axis and $M_{2}=3$ denotes the number along the *y*-axis).

**Figure 5:**
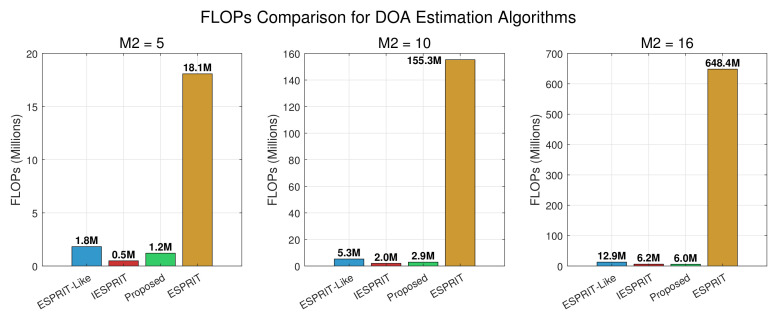
FLOP comparison of different algorithms.

Experiment 1: With the parameters set as $M_{1}=5$, $M_{2}=\left\{5,10,16\right\}$, K=3, and L=200, we investigate the computational complexity (in terms of FLOPs) of the considered algorithms under different array configurations. The comparison results are depicted in Figure 5. When $M_{2}=5$ and $M_{2}=10$, the proposed PM-based approach consistently ranks second in terms of computational load, slightly higher than IESPRIT but lower than ESPRIT and ESPRIT-like. However, as the array size increases, the trend shifts: at $M_{2}=16$, the computational advantage of the PM algorithm becomes evident, enabling it to achieve the lowest FLOPs among all methods. This outcome highlights the scalability of the proposed method, indicating that its efficiency gains are more pronounced for larger array configurations.

**Figure 6:**
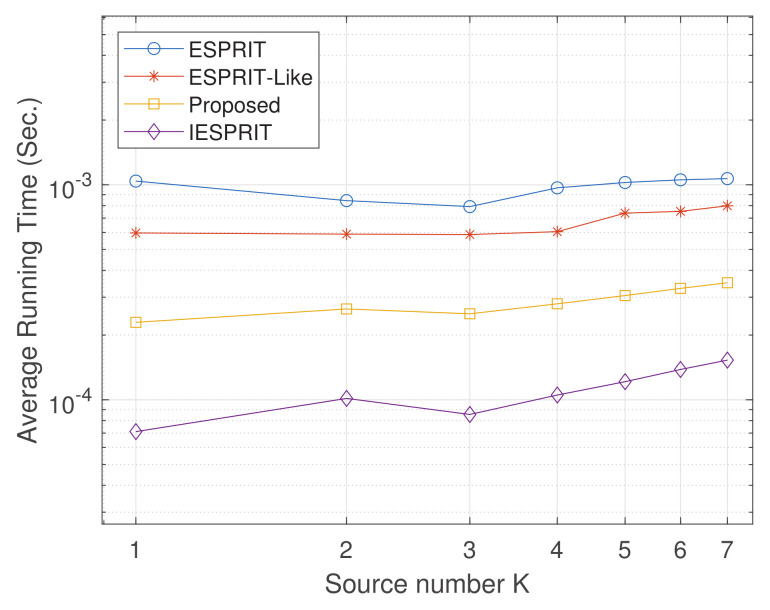
Comparison of average running time (ART) performance versus SNR.

Experiment 2: With parameters set as M=6, L=200, $d_{1}=2.5\lambda$, and $d_{2}=5\lambda$ (where $d_{1}$ denotes the spacing between array elements along the *x*-axis and $d_{2}$ denotes the spacing along the *y*-axis), we conducted 1000 Monte Carlo trials (item = 1000) to analyze the relationship between the average running time (AVT) and the number of sources *K*; results are illustrated in Figure 6. It is evident that the AVT of all algorithms remains relatively stable as *K* accumulates, with minimal fluctuations. Compared with classical ESPRIT and ESPRIT-like algorithms, both IESPRIT and the proposed method exhibit superior efficiency, attributed to optimized computational strategies. As shown in the figure, IESPRIT achieves the lowest average running time across all *K* values, while the proposed method outperforms ESPRIT and ESPRIT-like, particularly for K≥4. This improvement underscores the effectiveness of the enhanced algorithms in reducing computational complexity. Although IESPRIT achieves the lowest average running time, the proposed method still shows clear improvements over ESPRIT and ESPRIT-like by avoiding eigenvalue decomposition. This indicates that our method is efficient, though not the absolute minimum in runtime.

**Figure 7:**
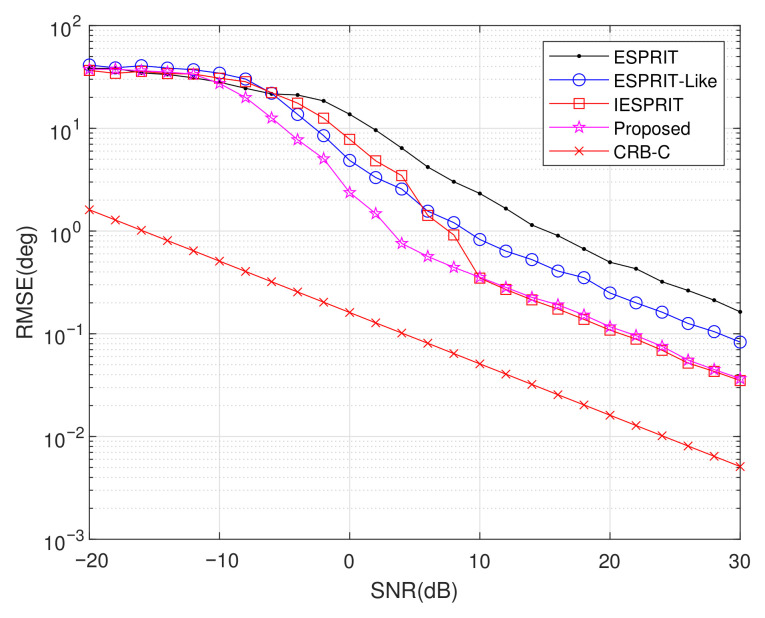
Comparison of RMSE performance versus SNR.

Experiment 3: Setting M=6, L=200, $d_{1}=2.5\lambda$, $d_{2}=5\lambda$, and item = 500, we assessed the DOA estimation performance by examining the RMSE against SNR, with the results shown in Figure 7. It is noticeable that all RMSEs decrease as SNR increases, indicating enhanced estimation accuracy under higher-SNR conditions. Compared with classical ESPRIT and ESPRIT-like algorithms, IESPRIT and the proposed method exhibit superior performance owing to improved signal subspace estimation. As shown in the figure, for SNR≥−10dB, the RMSE of the other algorithms is significantly higher than that of the proposed method. This improvement highlights the effectiveness and robustness of matrix completion and the PM-based subspace estimation.

**Figure 8:**
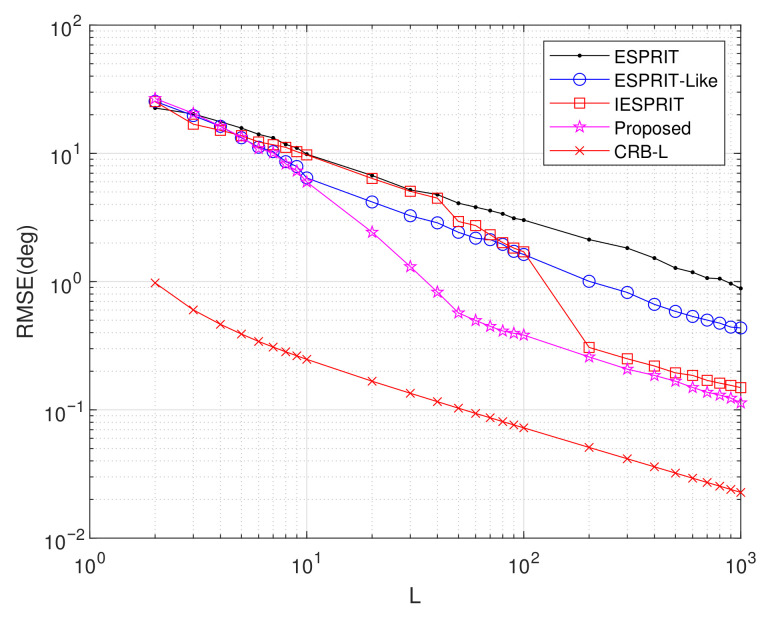
Comparison of RMSE performance versus *L*.

Experiment 4: Setting M=6, $d_{1}=2.5\lambda$, $d_{2}=5\lambda$, SNR = 10 dB, and item = 500, we evaluated the average RMSE performance as a function of sample size *L*, with results depicted in Figure 8. The plot reveals that all investigated algorithms achieve progressively lower RMSE values with increasing *L*. Throughout the tested range, the proposed methodology consistently surpasses alternative techniques in estimation accuracy. Particularly for L≥60, the proposed approach demonstrates substantial performance advantages, with its RMSE approaching the CRB as the number of snapshots increases. In contrast, competing methods exhibit a larger gap with this theoretical bound, indicating that the proposed technique provides improved effectiveness and robustness in delivering precise DOA estimates.

**Figure 9:**
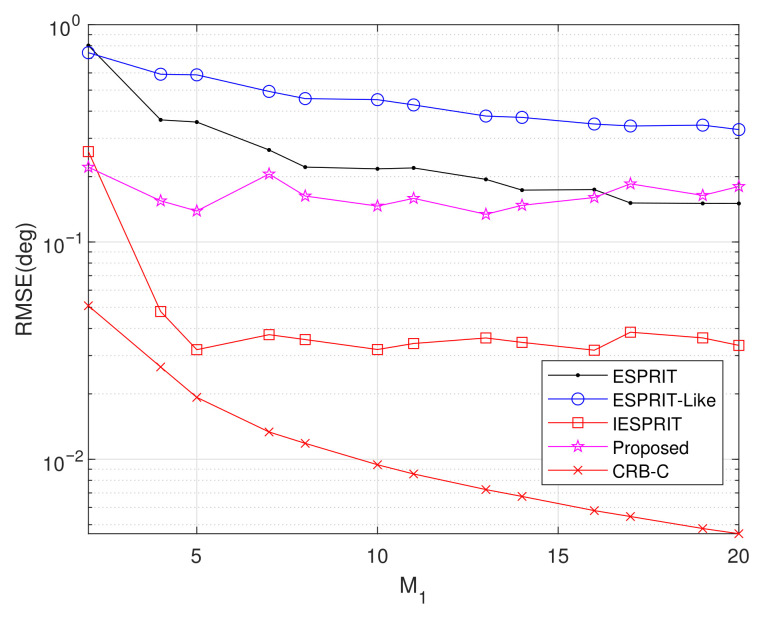
Comparison of RMSE performance versus $M_{1}$.

Experiment 5: Setting $M_{2}=3$, L=200, $d_{1}=2.5\lambda$, $d_{2}=5\lambda$, SNR = 10 dB, and item = 500, Figure 9 illustrates the average RMSE variation versus $M_{1}$. The CRB exhibits a monotonic decrease with increasing $M_{1}$ values, and both IESPRIT and the proposed method maintain performance advantages across the tested range. However, while the proposed algorithm demonstrates superior estimation accuracy when $M_{1}\leq 16$, ESPRIT achieves lower RMSE values for $M_{1}>16$.

**Figure 10:**
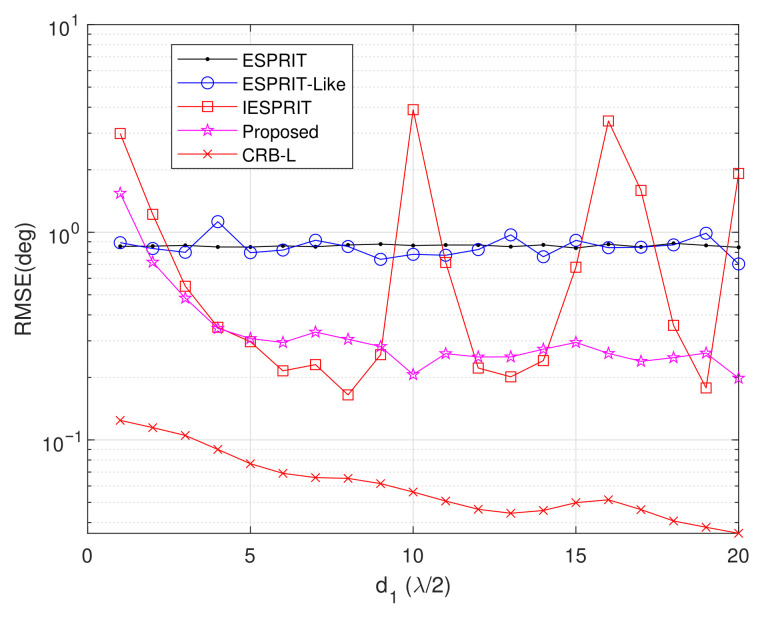
Comparison of RMSE performance versus $d_{1}$.

Experiment 6: Setting M=6, L=200, $d_{2}=5\lambda$, SNR = 10 dB, and item = 500, Figure 10 presents the average RMSE versus inter-element spacing $d_{1}$. This investigation focuses on element spacing variation in the L-shaped array configuration while maintaining fixed coprime array sensor positions. The results reveal a significant RMSE improvement for the proposed method with increasing $d_{1}$ values. However, beyond $d_{1}=10\lambda$, additional spacing increments yield diminishing returns in estimation accuracy, indicating performance stabilization. When $d_{1}>15\lambda$, the array aperture exceeds the Rayleigh distance, and wavefront curvature effects are introduced. This phenomenon violates the plane wave assumption, thereby limiting further reduction in RMSE. Notably, IESPRIT exhibits sporadic failures at specific spacing values, while the proposed algorithm maintains consistent robustness across all tested conditions. Furthermore, the proposed technique sustains an approximately 10 dB lower RMSE compared to ESPRIT and ESPRIT-like methods throughout the parameter range, demonstrating superior estimation accuracy and operational reliability.

**Figure 11:**
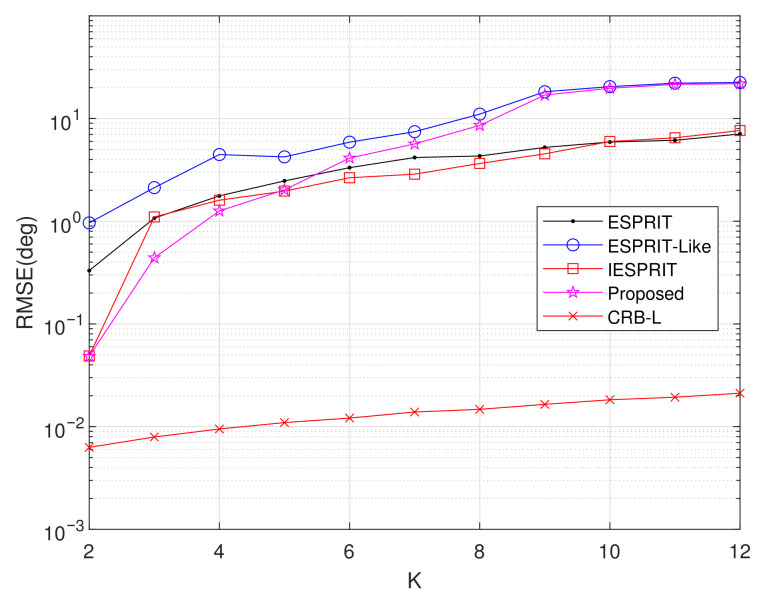
Comparison of RMSE comparison versus *K*.

Experiment 7: Setting M=6, L=200, $d_{1}=2.5\lambda$, $d_{2}=5\lambda$, SNR = 20 dB, and item = 500, this experiment investigates the influence of source count *K* on average RMSE performance, as illustrated in Figure 11. Results indicate a significant reduction in RMSE across all algorithms with increasing source count. For K≤5, the proposed method demonstrates significantly higher accuracy in comparison with benchmark algorithms. The performance advantage gradually diminishes when 6≤K<8, though our method remains dominant. At higher source counts (K≥8), while both IESPRIT and ESPRIT eventually surpass the proposed method, it consistently maintains a lower RMSE than ESPRIT-like methods.

### 5.2. Experiment Analysis

The proposed algorithm demonstrates significant advantages in suppressing nonuniform noise and achieving accurate DOA estimation under arbitrary array geometries. This can be visually confirmed in Figure 3 and Figure 4, where the scatter plots reveal that the proposed method effectively restores the source structure despite heavy noise contamination.

For comparison, several representative algorithms are considered. The ESPRIT algorithm is a classical method but is restricted to uniform or regularly structured arrays. The IESPRIT algorithm is known for its efficiency and exhibits high performance under sparse planar arrays; however, its reliance on structured deployment reduces flexibility in practical scenarios. The ESPRIT-like algorithm, which extends ESPRIT to arbitrary array geometries, serves as the primary baseline method. Although ESPRIT-like is efficient, the proposed method consistently outperforms it across all experiments. The analysis of simulation results is presented in Table 2.

In summary, the proposed method achieves a favorable balance between accuracy, robustness, and computational efficiency, making it a compelling choice for arbitrary array DOA estimation under nonuniform noise.

## 6. Conclusions

The present study presents a computationally efficient de-noising framework for 2D-DOA estimation using randomly spaced EMVS arrays in nonuniform noise environments. By leveraging the properties of nonuniform noise to construct the signal covariance matrix and eliminate noise-corrupted components, interference can be efficiently mitigated via low-rank matrix completion. Following this, the propagator method is utilized to retrieve the signal subspace, which circumvents the computational overhead associated with eigenvalue decomposition. Finally, ESPRIT-like processing enables rapid 2D angle estimation. Theoretical analyses confirm the algorithm’s high degrees of freedom, low computational complexity, and estimation accuracy. Furthermore, this method has promising applications in various fields, including radar systems, wireless communication networks, and remote sensing. In radar applications, it can be used for fast target localization and tracking, while in communication systems, it can assist in beamforming and direction finding for antenna arrays, improving signal quality and system performance in intricate environments.

## Figures and Tables

**Table 1:** Performance comparison among various methods.

Algorithm	Geometry	Flexibility	Identifiability	Complexity
ESPRIT	Sparse planar array	Low	min{M−1,6}	$6^{2}M^{2}L+10MK^{2}+6\left(M-1\right)K^{2}$ $+O\left(6^{3}M^{3}\right)+O\left(K^{3}\right)$
IESPRIT	Sparse planar array	Low	$\min\{\left(M_{1}-1\right)M_{2}-1,$ $\left(M_{2}-1\right)M_{1}-1\}$	$6^{2}M^{2}L+O\left(6^{2}M_{1}^{2}M_{2}^{2}K\right)$ $+O\left(6^{3}M^{3}\right)+O\left(K^{3}\right)$
ESPRIT-Like	Arbitrary	High	*M*	$6^{2}M^{2}L+10MK^{2}$ $+O\left(6^{3}M^{3}\right)+O\left(K^{3}\right)$
Proposed	Arbitrary	High	min{M,6}	$6^{2}M^{2}L+10MK^{2}+6\left(M-1\right)K^{2}$ $+O\left(6MKL+6MK^{2}\right)+O\left(K^{3}\right)$

**Table 2:** Simulation analysis summary.

Experiment	Scenario	Proposed Method Performance	Highlighted Property
Exp. 1 (Figure 5)	Computational complexity	Second ($M_{2}<15$), Best ($M_{2}>15$)	Efficiency for large arrays
Exp. 2 (Figure 6)	Average runtime	Second	Computational efficiency
Exp. 3 (Figure 7)	RMSE vs. SNR	Best	Robustness
Exp. 4 (Figure 8)	RMSE vs. L	Best	Robustness
Exp. 5 (Figure 9)	RMSE vs M_1_	second	Scalability and stability
Exp. 6 (Figure 10)	RMSE vs. d_1_	Best	Adaptability
Exp. 7 (Figure 11)	RMSE vs. K	Best (K < 5), second (K > 5)	Resolution capability

## Data Availability

Data is contained within the article.
